# Dupilumab-induced serum sickness-like reaction in a pediatric patient

**DOI:** 10.1016/j.jdcr.2024.11.013

**Published:** 2024-11-25

**Authors:** S. Minhaj Rahman, Fahad Ahmed, Carrington Webb, Adel Haque, Nicole Seminara

**Affiliations:** a College of Medicine, University of Rochester School of Medicine and Dentistry, Rochester, New York; b Perelman School of Medicine, University of Pennsylvania, Philadelphia, Pennsylvania; c Piedmont Plastic Surgery and Dermatology, Charlotte, North Carolina; d Dermatology Partners, Macungie, Pennsylvania; e Department of Medicine, University of New England College of Medicine, Biddeford, Maine

**Keywords:** atopic dermatitis, drug adverse effect, dupilumab, Dupixent, serum sickness

## Introduction

Dupilumab is a human monoclonal IgG4 antibody targeted against interleukins 4 and 13. It has been approved for atopic dermatitis (AD) since 2017, and in June 2022 dupilumab’s indication expanded to include patients of 6 months to 5 years of age.^1^ Dupilumab is generally well tolerated but there have been 4 case or clinical trial reports of classic serum sickness (CSS) or serum sickness-like reaction (SSLR) in adults.^2^, ^3^, ^4^ CSS is a systemic type III hypersensitivity reaction to foreign proteins in antitoxins, monoclonal antibodies, or antigenic vaccination resulting in immune complex depositions into synovial joints and parenchymal tissue. Common manifestations include pyrexia, urticarial rash, and polyarthralgia. In contrast to CSS, SSLR does not involve the formation of immune complexes. Because of a nonactivated complement system in SSLR, C3 and C4 complement levels are normal. To date, there have been no reported cases of CSS or SSLR due to dupilumab in the pediatric population. We present a case of SSLR reaction after dupilumab administration in a pediatric patient.

## Case description

**Fig 1:**
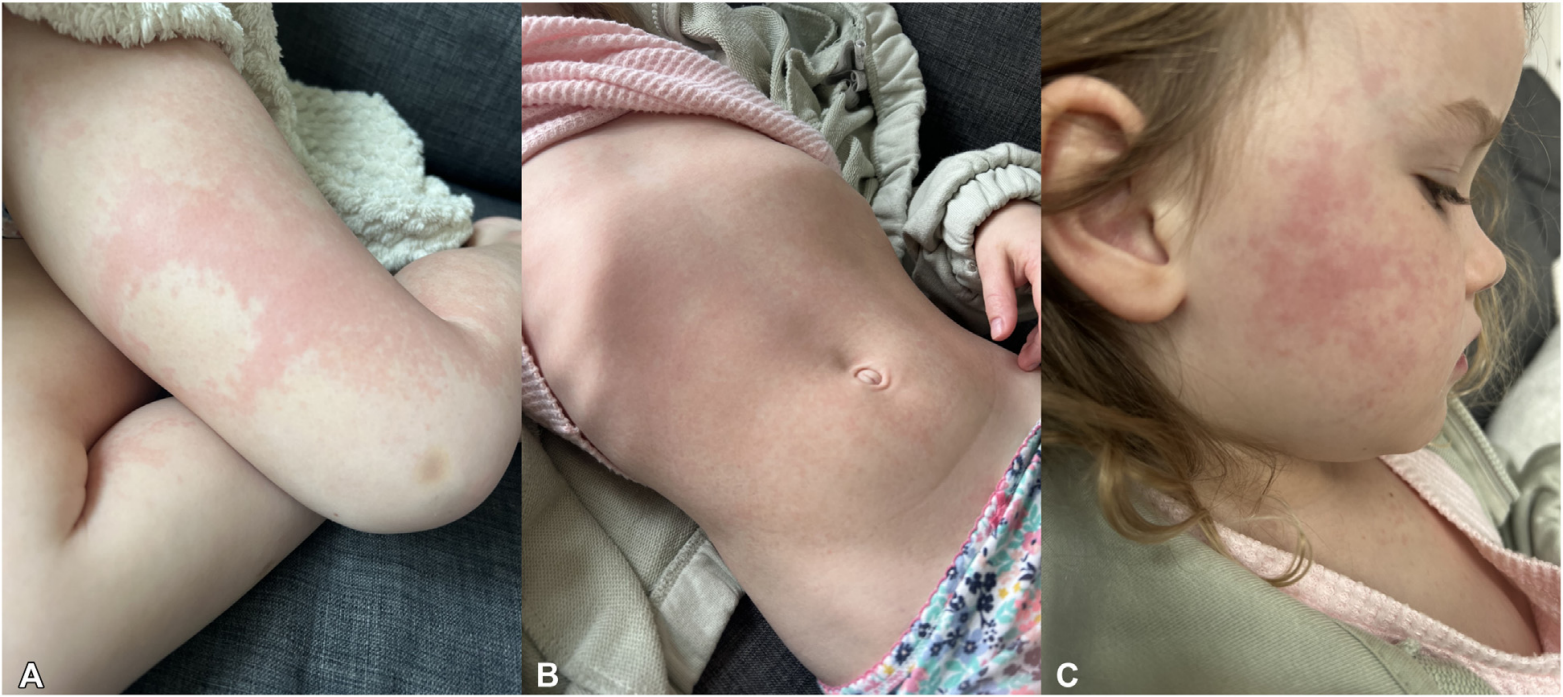
Red urticarial coalescing plaques around injection site of (**A**) upper portion of the left thigh, (**B**) spreading to her abdomen and (**C**) face.

**Fig 2:**
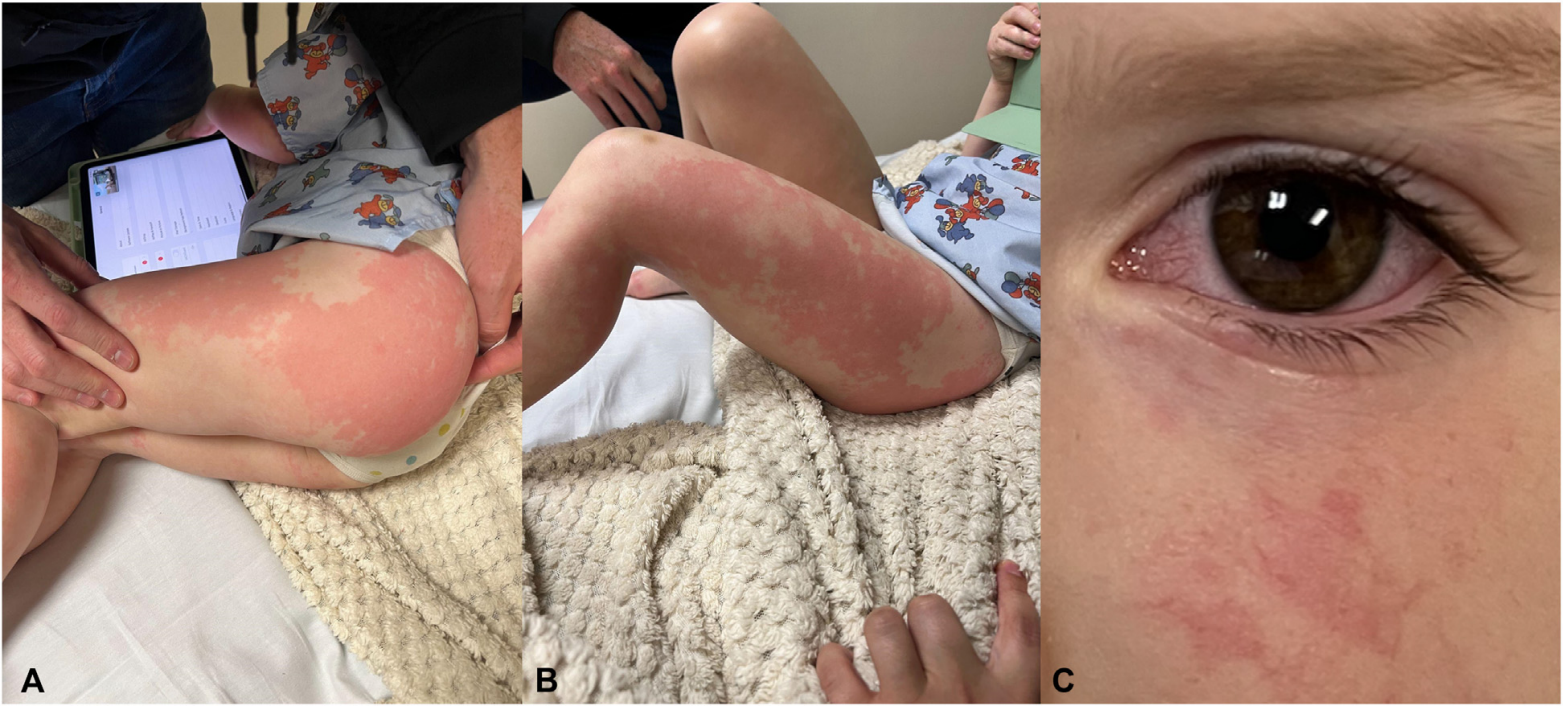
Worsening rash presentation in the emergency department depicting red urticarial plaques distributed across the (**A**) left buttock, (**B**) lateral side of the left thigh, and (**C**) injected conjunctiva.

A 3-year-old girl presented with a lifetime history of severe AD (SCORing Atopic Dermatitis Scale 58) not responsive to regular application of emollients, topical hydrocortisone, and topical triamcinolone. Because of disease severity, she was started on dupilumab 300 mg/2 mL every 4 weeks. The patient experienced an abnormally rapid resolution of her AD symptoms and was largely clear 9 days after injection. Sixteen days postinjection, she complained of left leg pain and her parents noted she was walking with a limp. Within 12 hours, red urticarial plaques developed around the injection site (Fig 1, *A*). Over the next 8 hours the rash rapidly expanded to include her face, both legs, arms, abdomen, and her buttocks (Fig 1, *B*, *C*). She was taken to an emergency department at which time her arthralgias had become so severe she was unable to walk and had to be carried. On arrival, she had a low-grade fever (102.0 °F) with injected conjunctivae, worsening rash, and no evidence of lymphadenopathy (Fig 2). Initial laboratory tests showed an elevated erythrocyte sedimentation rate (40 mm/h; <10) and C-reactive protein (5.22 mg/L, <5). Complement C3 (133 mg/dL, 86-166) and C4 (14 mg/dL, 13-46) were both within normal limits. Urinalysis was negative for proteinuria and her WBC count differential was negative for eosinophilia, lowering suspicions for drug reaction with eosinophilia and system symptoms. She was diagnosed with acute SSLR and was treated with prednisolone 18 mg 2 times daily for 7 days. Her rash, arthralgias, and fever resolved within 2 days of starting prednisolone. After acute SSLR resolution, there were extensive discussions about next steps for treating her severe AD however, this proved unnecessary as her baseline AD returned at a much lower level and she has not required further systemic treatment.

## Discussion

Theoretically, all monoclonal antibodies pose the risk of inducing SSLR. The suggested pathophysiology of CSS involves exposure of a foreign protein to which the body develops antibody-antigen complexes that deposit into the synovial joint fluid and parenchymal tissue approximately 7 to 21 days after foreign exposure. SSLR pathogenesis, however, is not well understood but defined as a similar clinical manifestation as CSS without immune complex formation. Common manifestations for both CSS and SSLR include urticaria (typically first noticed at the site of injection), cutaneous eruptions, fever, myalgias, arthralgias, and lymphadenopathy. More severe cases of CSS can present with leukocytosis, thrombocytopenia, proteinuria, or hematuria. However, these symptoms, indicative of multisystem organ involvement, are far less likely in SSLR and its absence in our patient make SSLR more likely.^5^ Further, CSS immune complex deposition typically activates the classical complement pathway leading to decreased circulating C3 and C4 levels. As SSLR does not involve immune complement deposit, normal C3 and C4 complement levels can be used to differentiate between CSS and SSLR, as presented in our patient.^5^ Therefore, our patient’s presentation of fever, myalgias, arthralgias, classically distributed rash, elevated erythrocyte sedimentation rate and C-reactive protein, absence of multisystem organ involvement, and normal C3 and C4 complement levels 16 days after her first dose of dupilumab is highly consistent with SSLR. After SSLR, our patient displayed a rapid resolution of symptoms and has sustained improvement after years of having severe AD. Because of this change, the authors invite the possibility of lasting immunologic effects from SSLR.

Because of its self-limiting nature, treatment is largely drug cessation and supportive care including corticosteroids, antihistamines, and antipyretics.^6^ The prevalence of SSLR is unknown as it is rarely reported and may be mistaken for other drug induced cutaneous manifestations.^7^ During the clinical trial development of dupilumab, administrators noted one adult with CSS and another with SSLR, both being treated for AD. Since then, 2 additional adult cases have been reported in the literature, one patient who was treated for aspirin-exacerbated respiratory disease and allergic rhinitis, and another case treated for AD.

Jung et al^4^ reported a case of a 29-year-old woman, who experienced an immediate type I hypersensitivity reaction to dupilumab after first exposure, presenting with diffuse urticaria and asthma exacerbation.^4^ After a rechallenge 2 years later, she experienced symptoms indicative of CSS including myalgia, facial and hand swelling, arthralgia, rash at the injection site, and elevated inflammatory markers within 1 to 3 days, likely due to prior sensitization. Although the presented pediatric patient did not require additional dosages of dupilumab, the development of CSS and SSLR in this adult patient after initial medication sensitization can be helpful in assessing future pediatric prognosis in those who may consider additional dupilumab administration. Treudler et al^3^ reported a case of a 54-year-old man with severe AD who experienced SSLR 10 days after his initial dupilumab dose with symptoms resurfacing after early administration of his second dose on day 12. Although researchers identified 2 epitopes on dupilumab’s heavy chain where antidrug antibodies were bound, suggestive of SSLR, they also discovered undetectable IgA levels. Given that autoimmune diseases, including CSS, have been associated with primary immunodeficiencies, this raises concerns about IgA deficiency as a potential predisposing factor for SSLR.^3^^,^^8^ Understanding this suggested association in adult patients may provide insights for future pediatric prognosis.

As potential adverse effects of monoclonal antibody therapy are further classified, additional studies specifically elucidating the significance and linkage between therapy and immune reactions are necessary. As the usage of dupilumab becomes more prevalent in the pediatric population, documenting adverse reactions such as SSLR in this population becomes paramount for clinicians to initiate timely and appropriate patient management.

## Conflicts of interest

Dr Haque is a speaker for Regeneron, Sanofi, AbbVie, Amgen, Sun Pharmaceuticals, Eli Lilly, Johnson and Johnson, and Dermavant Sciences.

Dr Seminara is a speaker for Abbvie, Pfizer, Johnson and Johnson, Eli Lilly, Galderma, Dermavant Sciences, Sun Pharmaceuticals and UCB.
